# Safety and Efficacy of Initiating Parenteral Nutrition at Home, Home Start PN, in Advanced Peritoneal Metastasis

**DOI:** 10.3390/cancers16244272

**Published:** 2024-12-23

**Authors:** Chunmeng Zhang, Ujwal Yanala, Mounika Addula, Sherry Adams, Louise Ocken, Patricia Skiendziel, Tia Bodkins, Jason M. Foster

**Affiliations:** 1 Departments of Surgery, Surgical Oncology, University of Nebraska Medical Center, Omaha, NE 68198, USA; 2 Department of Internal Medicine, Creighton University Medical Center, Omaha, NE 68124, USA; 3 Nutrition Support Team, BioScrip Infusion Services, Omaha, NE 68127, USA

**Keywords:** parenteral nutrition, TPN, advanced oncology patients, home start TPN, safety, efficacy

## Abstract

Background: Patients with peritoneal carcinomatosis often experience intestinal failure throughout the course of their disease, and total parenteral nutrition (TPN) can be used as a temporary solution or as a bridge to definitive cytoreductive surgery. Guidelines for TPN are well established for inpatients and in 2014, guidelines were established for the initiation of TPN for outpatients in a home setting. However, the safety and efficacy of home start TPN in advanced oncology patients remain unknown. This study aims to explore the safety and efficacy of starting TPN in the home setting for patients with peritoneal carcinomatosis. Method: Health records of advanced cancer patients receiving TPN during 2009–2020 were retrospectively reviewed. Data pertaining to diagnosis, demographics, nutritional parameters, and outcomes including hospital readmission rates were collected. Safety was measured based on catheter-related complications and hospital admissions related to electrolyte or fluid imbalance due to TPN. Efficacy was determined by weight gain/stability and pre-albumin and albumin levels. The Fisher’s exact and Kruskal–Wallis tests were used to analyze the data. Results: Seventy TPN patients were identified, of which forty-two were home start (HS) and twenty-eight were in hospital (HP). The two groups were not significantly different in age, (HS: mean = 58.3 ± 13.9; HP: mean = 58.0 ± 13; *p* = 0.95), baseline body weight (*p* = 0.13), baseline albumin (*p* = 0.26) or pre-albumin (*p* = 0.48). At the end of treatment, the HS and HP groups had similar percentages of patients experiencing weight gain/stability (75% vs. 47%, *p* = 0.1), stable/increased pre-albumin (68% vs. 65%, *p* = 1), and stable/increased albumin levels (48% vs. 59%, *p* = 0.58). There was no difference in observed readmission between the groups (*p* = 0.79). At the end of treatment, 48% of the HS group and 36% of the HP group resumed an oral diet. Conclusions: This is the first study to present a comparison between home and hospital start TPN in advanced cancer patients, demonstrating that the initiation of outpatient TPN in the home setting is as safe and efficacious as TPN initiated in the hospital.

## Simple Summary

In this study, we report on the safety and efficacy of initiating parenteral nutrition in the home setting (HomeStart PN) for patients with advanced peritoneal metastasis. This analysis provides data demonstrating that initiating TPN at home is as safe as initiating TPN in a hospital setting. In our retrospective review, we compared outcomes of patients who initiated TPN at home and those who was admitted to the hospital for acute conditions related to their cancer and started TPN who otherwise would have qualified for Home Start PN. These two groups had similar baseline weight and nutrition status prior to starting TPN, and following TPN nutritional support, achieved comparable weight gain/ weight stability and achievement of nutrition resuscitation goals and stability. The clinical course analysis revealed 35–45% of patients with advanced peritoneal metastasis experience hospital admission/readmissions due to the underlying cancer and therapies to treat disease for both home- and hospital-initiated TPN. Given the frequent hospitalizations for this patient population, a Home Start PN approach in this advanced oncologic patient population is advantageous and potentially reduces time spent in hospital and the associated costs.

## 1. Introduction

Patients with peritoneal carcinomatosis often experience intestinal failure throughout the course of their disease, some at presentation, some in the middle, and some towards the end [1]. Mechanisms of intestinal failure in these patients include factors directly or indirectly related to their tumors or prior to treatment, such as mechanical obstructions (extrinsic bowel compression, adhesion, intraluminal obstruction, anastomotic/ischemic strictures, radiation enteritis/fibrosis) and/or functional disorders (tumor infiltration of bowel wall or autonomic nerve plexuses, drug-induced ileus, post-op ileus) [2]. These patients experience frequent admissions for bowel dysfunction, which negatively impacts their quality of life and result in a significant financial burden. Parenteral nutrition is commonly used to support patients with enteral failure so they can continue to pursue systemic chemotherapy, surgery, and improve their quality of life. Although there are ASPEN guidelines for “home start PN”, due to the paucity of data, inpatient initiation of TPN remains the primary model for starting parenteral nutritional support [3]. There is a need to provide data that establishes the safety of initiating parenteral nutrition in the ambulatory setting, which can have a positive impact on this patient population, particularly by avoiding unnecessary hospitalizations, improving patient quality of life, and providing a bridge to medical therapy and/or surgical therapy that can resolve obstruction and address symptoms.

Parenteral nutrition is defined as the intravenous administration of simple macronutrients (amino acids, dextrose, lipids) given as a balanced combination of sterile nutrients. This supply of nutrients could either be complementing nutritional intake (in cases where the patients are nutritionally deprived but able to consume clear liquids for comfort) or complete (where patients are incompetent of food/nutritional intake via the alimentary tract). Total parenteral nutrition (TPN) has revolutionized nutritional management and increased survival in patients with malabsorption and malnutrition. Early implementation of TPN in benign conditions has improved survival in patients challenged by malnutrition. Additionally, TPN support has been utilized in cancer patients with nutritional deprivation secondary to cachexia and has yielded similar benefits [4,5].

Throughout the years, as clinicians have gained more experience, guidelines have been established and optimized for the proper administration of parenteral nutrition. Current indications for the administration of TPN range from inadequate absorption with short bowel syndrome, gastrointestinal fistula leading to large-volume enteric nutritional losses, prolonged bowel obstruction, the need for prolonged bowel rest, severe malnutrition/failure to thrive, and other disease states where oral or enteral nutrition is not possible. Over the past 50 years, the use of parenteral nutrition has undergone changes in administration rates, patient setting, and patient population. This evolution has made its utilization more acceptable and ubiquitous [6]. The most common strategy for TPN initiation is to admit the patient to hospital, obtain central access as an inpatient and administer the index TPN formulation while closely monitoring for complications such as electrolyte abnormalities and catheter-related issues. The initiation of parenteral nutrition at home has become an option more recently [7,8], and the American and European Societies of Parenteral Nutrition have developed guidelines for patient selection and safety. Specifically, home start PN is recommended for patients who demonstrate stability in their overall health and are not at high risk of refeeding syndrome [9,10]. While Inayet et al. provide a commentary on the safety and rigor of outpatient-initiated PN, there is paucity of clinical data demonstrating the practical safety of home-initiated parenteral nutrition [6]. The aim of our study is to demonstrate the safety and efficacy of outpatient parenteral nutrition started in the home setting for patients with peritoneal disease compared to a group of similar patients who started TPN in hospital. We measured rates of complications and nutritional parameters in the two groups to establish that TPN at home is as safe as TPN in hospital and achieves the same level of nutritional support in an advanced oncology population.

## 2. Methods

Patients receiving TPN between the periods of January 2009 and April 2015 managed by one home health and infusion agency, and a single provider at our institution were studied. Specifically, all patients in the study had advanced gastrointestinal cancer, primarily with peritoneal metastatic disease or complex primary gastrointestinal malignancies.

De-identified data were provided by the home health agency and an IRB waiver was obtained for the utilization of this de-identified data. Unique identifier codes were generated, and subjects were classified into two groups: the home start (HS) group where patients had outpatient central venous access obtained as an ambulatory procedure and parenteral nutrition was initiated in the home setting; the hospital start (HP) group where patients were admitted to the hospital, central venous access was placed and parenteral nutrition was initiated and titrated appropriately to normalize nutritional abnormalities while in hospital. HP patients were then transitioned to their homes after obtaining stability. All of the patients had similar baseline health and indications for parenteral nutrition.

### 2.1. Patient Selection for Home Start TPN

Our established criteria to start TPN at home, adapted from ASPEN, focused on ensuring the absence of high-risk features that place patients at high risk of known complications. The patients needed to meet the following criteria:

1. No significant renal failure
2. No significant congestive heart failure
3. No significant liver failure
4. No poorly controlled diabetes
5. Presence of working central venous access device
6. Intact volume status
7. Patient and family able to understand instructions
8. Not at high risk of refeeding (relative contraindication)

All patients in the HS group were screened prior to starting TPN at home, and none were at risk of refeeding syndrome, as per ASPEN guidelines [11]. The detailed guidelines and protocol for initiating and monitoring home TPN can be found in Table 1. Briefly, baseline labs were obtained which included complete blood count (CBC), comprehensive metabolic panel (CMP), magnesium, inorganic phosphate, pre-albumin, and triglycerides, prior to starting TPN. Any electrolyte or volume deficiencies were corrected at home.

### 2.2. Initiation Process for TPN at Home

After satisfying the patient selection criteria according to the pre-established requirements, this selected group of patients underwent a protocol-based initiation of parenteral nutrition at home after outpatient peripherally inserted central (PICC) catheter placement. This protocol included:

1. Initiation at a 24 h infusion rate.
2. Use of 2:1 or 3:1 solution containing 100% of protein needs, 50% of glucose needs, 75–100% of volume needs and 75–100% of lipid needs.
3. Repeating basic metabolic panel (BMP), magnesium and phosphorus laboratory investigations for three consecutive days after TPN initiation.
4. After achieving stable laboratory findings for the initial 3 days, patients were transitioned to bi-weekly laboratory checks as follows:

CBC, CMP, magnesium, phosphorus, triglycerides, and pre-albumin on Mondays and BMP, magnesium, and phosphorus on Thursdays.

### 2.3. Hospital Start TPN Transitioned to Home

This was a similar group of patients admitted with an acute condition related to the underlying cancer who otherwise would have been candidates for the HS group. PICC central venous access was placed in an inpatient setting and TPN was initiated and adjusted for at least the first 3 days while the patients were hospitalized and recovering from acute illnesses. After discharge, patients were then transitioned to outpatient TPN management.

### 2.4. Data Collection

This retrospective study included patients receiving TPN between January 2009 and April 2015 at our institution whose parenteral nutrition was managed by a single home health/infusion agency to avoid confounding data. Seventy patients were identified, of which 42 were in the HS group and 28 were in the HP group. Age, sex, primary diagnosis, and indication for TPN were provided for each patient. Nutritional variables collected included weight and albumin and pre-albumin levels. Values were measured at the initiation of therapy, bi-weekly throughout the duration of therapy, and at the termination of therapy. The duration of TPN was measured for each patient. Weight, albumin, and pre-albumin stability/increase as well as the overall change in these nutritional metrics were compared between each group. Similarly, the safety of the HS group vs. the HP group was explored by measuring readmission rates, TPN electrolyte levels, dehydration levels, and fluid overload events both at initiation and throughout the duration of TPN. Mortality and readmission rates were measured for the HS and HP groups at 30 days and from 31 to 90 days. The overall rate of readmission was determined and categorized into four groups: (1) directly related to TPN; (2) central-line related, (3) planned admission for surgery, chemotherapy, or any other planned intervention requiring hospitalization; and (4) unplanned admission for underlying illnesses, acute illnesses, or complications unrelated to planned admissions.

### 2.5. Statistics

Because the two groups (home start vs. hospital start) were of unequal sample sizes (42 vs. 28), the Kruskal–Wallis statistical test was used for analysis to compare age, weight, and albumin and pre-albumin levels. Fisher’s exact test was used to compare and analyze readmission rates. Results were statistically significant for a *p* value of 0.05 or less.

## 3. Results

### 3.1. Demographics and Baseline Parameters

Eighty-nine percent (90% HS, 89% HP) of patients had peritoneal metastasis and all patients had abnormal albumin and pre-albumin levels prior to starting TPN, as shown in Table 2. There was no difference in median age (HS: 58 vs. HP: 59, *p* = 0.95) or sex: 45% (19/42) of the HS group were female, which was not different from the HP group, where 54% were female (15/28). Baseline weight, albumin, and pre-albumin levels were similar between HS and HP, as shown in Table 3, and the observed median (55 vs. 45 days) and average (82 vs. 82 days, *p* = 0.9512) duration of TPN therapy for HS and HP were comparable between the groups as well.

### 3.2. Safety Parameters

Thirty-day admissions were 43% (18) in the HS group and 39% (11) in the HP group (*p* = 0.68). Readmission rates at 31–90 days were 29% (12) and 18% (5) (*p* = 0.80) for HS and HP, respectively. Total 90-day readmissions were 55% (23) for HS and 46% (13) for HP, *p* = 0.63. There were no readmissions directly related to home TPN in either group. There were two CLABSI in HS, and one provoked DVT in HP due to central access that resulted in 30-day readmission. All other admissions/readmissions were due to underlying disease, as shown in Table 4.

### 3.3. Efficacy Parameters

Baseline values of weight, albumin and pre-albumin levels were similar for each group (Table 1), and no demonstrable difference was observed in objective nutritional parameters (Table 4). Specifically, 75% of the HS group demonstrated improved or stable weight at the end of treatment compared to 47% in the HP group (*p* = 0.1). A total of 48% of the HS group had stable/improved albumin compared to 59% of the HP group (*p* = 0.6), while 68% of the HS group and 65% of the HP group (*p* = 1.0) showed stable/improved pre-albumin levels. When improvement occurred, the average increase in albumin and pre-albumin levels was 18% and 52% in the HS group with an average weight increase of 9%. In the HP group, among the patients who showed improvement, average changes in albumin levels, pre-albumin levels and weight were 14%, 71% and 4.5%, respectively. At the end of treatment, 48% of the HS group and 36% of the hospital start group resumed an oral diet (Table 3).

## 4. Discussion

Parenteral nutrition has revolutionized the management of patients unable to tolerate oral/enteral nutrition or able to tolerate PO/enteral nutrition but unable to achieve and sustain daily nutritional goals. A large body of literature exists, spanning children with necrotizing enterocolitis to adults with inflammatory bowel disease, intractable fistulas, and cancer [4,5]. The protocol of initiating parenteral nutrition in hospital, with optimization for 5–10 days, and transitioning to home has been well established as a safe and effective model for patients requiring parental nutritional support [12,13]. Although ASPEN has established and promotes criteria for outpatient home start TPN [3], the majority of stable patients who only need nutritional support are still admitted to hospital to initiate TPN due to the absence of data validating the safety of starting TPN at home. This study demonstrates that the safety of home start TPN is comparable to hospital-initiated TPN for patients with peritoneal carcinomatosis. During this retrospective study period, 60% of the TPN was initiated in the home setting with no observed TPN complications. Catheter-related complications were the only indirect cause of TPN 30-day admissions and occurred at similar rates in the HS and HP groups. Specifically, there were two CLABSI in the HS group and one PICC-associated VTE in the HP group. These rates are comparable to central-line-related readmissions in the literature. Overall, the rate of catheter-associated DVT was 1.4% (1/70) and the CLABSI rate was 2.8% (2/70). Cotogni et al. prospectively observed 250 patients, of whom 98% had solid malignancy needing home parenteral nutrition with PICC lines as their central venous access. As reported in the study, the incidence of catheter-related bloodstream infections was 2.2% (0.05 per 1000 catheter days), and the incidence of PICC-related symptomatic thrombosis was 1.1% (0.05 per 1000 catheter days). The overall complication rate was 17.5% (0.85 per 1000 catheter days) and PICCs were removed because of complications only in 7% of cases, thus establishing that PICC line use is safe in the home setting [13]. A second study confirmed the safety of PICC and demonstrated the same level of CLABSI and DVT as ports [12]. Similarly, PICC-associated complications in this study were consistent with those reported by Cotogni et al. and other published studies [14,15,16,17,18,19,20,21].

Electrolyte abnormalities and refeeding risks have been the major concerns driving hospital initiation of TPN. Risk factors for refeeding syndrome include severe malnutrition with a BMI < 18.5 kg/m^2^, unintentional weight loss > 10% within 3–6 months, very little or no food intake for >5 days, and a history of alcohol misuse or drugs, including insulin, chemotherapy, antacids, or diuretics, and should be considered relative contraindications to starting TPN at home [22,23]. In our study, asymptomatic abnormal electrolytes occurred in 19% of home start patients during the entire course of parenteral nutrition that were safely corrected at home or at an outpatient infusion center with appropriate IV fluid electrolytes. In some cases, they were corrected with adjustments to the TPN formulation alone. To our knowledge, this is the first study evaluating the safety of initiating TPN at home and demonstrating the low risk of refeeding syndrome after careful patient selection. In addition to optimal patient selection, other strategies to prevent refeeding syndrome include repletion of fluid and electrolytes prior to starting TPN, using a low-carbohydrate, moderate-volume solution and advancing the infusion slowly. Frequent monitoring of electrolytes and timely repletion after initiating TPN is also central to the safety and success of home start TPN [8]. Lastly, as described by Inayet et al., paramount to safe outpatient TPN initiation is an experienced nutrition support team that includes home health nurses, pharmacists and a certified nutrition support clinician/registered dietitian [7,8]. This team is essential for the close monitoring of electrolytes, rapid identification of abnormalities, efficient preparation and administration of electrolyte replacements, as well as ensuring the optimal nutritional formulation for each patient. This is consistent with the existing literature regarding the transition from hospital to home TPN for optimal safety where an experienced nutrition support team can optimize electrolyte abnormalities and reduce 30-day mortality [24]. Patient and family education, including instruction on TPN infusion and recognition of the signs and symptoms of catheter-associated complications and electrolyte abnormalities, also plays an important role in the safety and success of home TPN [25]. A few studies have reported hypersensitive reactions to TPN, but none were observed in our study [26].

The American Society for Parenteral and Enteral Nutrition (ASPEN) has established guidelines and consensus recommendations on TPN in hospitals, skilled nursing facilities, long-term acute care hospitals, rehabilitation facilities, and for safe initiation in the home setting [3,10,27]. ASPEN has been promoting the use of parenteral nutrition when necessary and appropriate to provide nutritional requirements in malnourished and debilitated patients. Utilizing ASPEN guidelines, 42 patients who satisfied the criteria for initiation of TPN at home (Table 1) were safely started in the home setting after a PICC was placed in the ambulatory setting prior to or on the same day TPN was initiated at home. There were no complications associated with the outpatient placement of central access or related to the TPN started at home. Readmissions in both the home start and hospital start groups were driven by underlying diseases and medical conditions that catalyzed the need for TPN. At the end of treatment, almost half of the HS group resumed an oral diet, further illustrating not only the safety but also the efficacy of TPN in this patient population as a temporary solution to facilitate their recovery from intestinal failure due to treatment/surgical complications and as a bridge to their definitive treatment success.

## 5. Limitations

The major limitation of this study was the retrospective design and level of illness in this group of cancer patients with other drivers of cachexia. The hospital start (HP) group provided a control group to account for this limitation, and there was no significant difference between these groups in weight, albumin, and pre-albumin metrics. By following ASPEN guidelines for monitoring the nutrition endpoint, this study demonstrated that 75% of home start patients maintained or gained weight with an average gain of five pounds. As the data were retrospective, it was difficult to assess whether the weight stability or increase was secondary to preserved/improved muscle mass; however, none of the patients had clinically significant edema or ascites. All of the patients were regularly monitored using imaging for cancer surveillance every 3 months. Similarly, albumin and pre-albumin levels were maintained or increased in the majority of patients. Most importantly, the observed changes in the home start group were the same as in the hospital start group, which represented a matched control group.

Another limitation of the study was the small sample size, which limited its statistical power. The results of this study should be interpreted with caution because both type I and type II statistical errors could exist. Specifically, while no statistically significant difference was observed in terms of baseline characteristics and post-treatment changes between the two groups, the HP group has a trend towards higher body weight (157 vs. 141 lbs, *p* = 0.13) compared to the HS group. It has been previously demonstrated that a higher baseline body weight is associated with prolonged survival in cancer patients on home parenteral nutrition [28], and because the HP group can be assumed to include sicker patients with more comorbidities or acute illness requiring hospitalization, this trend of higher baseline body weight would contribute towards a type II error and support our conclusion that HS TPN is as safe and effective as a hospital start strategy. Similarly, after the TPN course, although there is no significant difference between weight gain, the HS group has a trend towards higher weight gain (75% vs. 47%, *p* = 0.1), again this could reflect a type II error due to the small sample size and supports our conclusion.

To address these limitations, future work should include recruiting additional patients to increase the statistical power of the study, providing both internal and external validation for our findings. Preferably, this should be done in a randomized controlled trial format to minimize other confounding factors influencing the study results.

## 6. Conclusions

In summary, parenteral nutrition can be safely initiated at home in patients with advanced cancers who are nutritionally deficient, not acutely ill, and not candidates for enteral support.

This is the first study demonstrating the safety of home PN initiation and the additional value of avoiding hospitalization in advanced oncology patients who are inherently at a high risk of hospitalization due to their underlying cancer. Careful monitoring, an experienced home health infusion nutrition support team, and a compliant and competent patient are integral to the successful initiation of TPN at home. Starting TPN during the early phases of a complex disease process, including metastatic gastrointestinal malignancies, can not only improve survival but also improve the quality of life of these patients.

## Figures and Tables

**Table 1:** Criteria for patients to initiate PN at home, process of initiation, monitoring and addressing risk factors such as refeeding syndrome.

Patient selection for Home Start (HS) PN:
No significant renal failure
No significant congestive heart failure
No significant liver failure
Diabetes under good control
Presence of working central venous access device
Intact volume status
Fully able to understand instructions
Not at risk of refeeding (if developing refeeding syndrome, follow additional guidelines below)
**Laboratory Investigations Prior to Initiation of PN:**
CBC (complete blood count with differential)
CMP (glucose, electrolytes, calcium, blood urea nitrogen, creatinine, liver function tests),
Triglycerides, pre-albumin and other electrolytes like magnesium and phosphorus
**PN Initiation at Home:**
Initiate at 24 h cycle
Use 2:1 or 3:1 solution containing 100% of protein needs, 50% of glucose needs, 75 to 100% of volume needs, and 75 to 100% of lipid needs
Repeat BMP, magnesium and phosphorus for 3 days consecutively after PN initiation
After achieving stable laboratory findings for initial 3 days, transition to biweekly labs as follows: CBC, CMP, Mg, phosphorus, pre-albumin on Mondays; BMP, Mg and phosphorus on Thursdays
Weight monitored weekly
**PN Additional Guidelines for Patient who developed Refeeding Syndrome**
Consider 3-day infusion of 100 mg thiamin
Limit volume to 1 liter at initial initiation
Limit carbohydrate to 100–150 g
Limit protein to 50–75%
Limit lipids to 50–100%

**Table 2:** Demographic data showing number of cases and controls, their clinical diagnosis, median age, nutritional parameters prior to initiation of PN in the two groups including weight, albumin and pre-albumin. IQR, interquartile range. NOS, not otherwise specified.

Variables		HS Group (#)	HP Group (#)	*p* Value
		42	28	
Clinical diagnosis—PM primary	Cancer type			
	Appendix	9	4	
	Pancreas	2	2	
	Stomach	5	0	
	Small bowel	2	2	
	Colon	10	9	
	Peritoneal carcinomatosis, NOS	6	3	
	GU tract	4	2	
	Breast/other metastatic dis.	2	0	
	Others	2	6	
	TOTAL	N = 42	N = 28	
Median age (IQR, years)		58 (52, 67)	59 (53, 65)	0.9526
Median weight (IQR, lbs.)		141 (123, 164)	157 (135, 184)	0.13
Median albumin (IQR, mg/dL)		2.9 (2.3, 3.5)	3 (2.7, 3.3)	0.2619
Median pre-albumin (IQR, mg/dL)		13 (8.5, 17.9)	15 (9.0, 20.4)	0.4785

**Table 3:** Description of treatment-related changes, improvement in nutritional parameters. IQR, interquartile range.

Variable Observed	Home Start	Hospital Start	*p* Value
% observed stable/increase			
Weight	75%	47%	0.1
Albumin	48%	59%	0.58
Pre-albumin	68%	65%	1
Average change observed			
Weight (lbs.)	5.6	−2.3	
Albumin (mg/dL)	0.02	−0.07	
Pre-albumin (mg/dL)	3.1	3.6	
Average duration of TPN (days)	82.05	81.96	0.9512
Median duration of TPN (IQR, days)	45 (27, 99)	55 (26, 114)	
Disposition at end of therapy			
Resume PO diet	20 (48%)	10 (36%)	
Still on TPN	5 (12%)	0 (0%)	
Deceased	15 (36%)	12 (43%)	
Hospice/Unknown	2 (5%)	6 (21%)	

**Table 4:** Readmissions following initiation of TPN. CRBSI—Catheter Related Blood Stream Infection. CVA: Cerebrovascular Accident. SBO: Small Bowel Obstruction. Decline indicates worsening disease progression leading to failure to thrive and progressive weakness. DVT: Deep Venous Thrombosis. MI: Myocardial Ischemia. AMS: Altered Mental Status. PE: Pulmonary Embolus.

	HS Group (N = 42)	HP Group (N = 28)	*p* Value
**Readmission within 30 days**	N = 18	N = 11	*p* = 0.6748
	CRBSI (2)	DVT (1)	
	CVA (1)	Abscess (2)	
	SBO (3)	Fever (2)	
	Decline (3)	Ascites (2)	
	Anemia (1)	MI (1)	
	Fever (2)	Sepsis (1)	
	Fistula (1)	SBO (1)	
	Seizures (1)	Ileus (1)	
	Cellulitis (1)		
	SOB (2)		
	Pain (1)		
**31–90 days**	N = 12	N = 5	*p* = 0.8012
	Decline (2)	Decline (1)	
	Fever (2)	Fever (1)	
	Fistula (1)	Pain (1)	
	Pain (4)	Pleural effusion (1)	
	Dumping (1)	GI bleed (1)	
	AMS (1)		
	PE (1)		

## Data Availability

Data described in the manuscript, code book, and analytic code will be made available upon request.
